# Evaluating the utility of storage solutions to preserve nucleic acid integrity at ambient temperature in South Africa

**DOI:** 10.4102/ajlm.v15i1.2915

**Published:** 2026-08-21

**Authors:** Timothy Moshoma, Maemu P. Gededzha, Nakampe Mampeule, Elizabeth Mayne

**Affiliations:** 1 Department of Immunology, School of Pathology, Faculty of Health Sciences, University of the Witwatersrand, Johannesburg, South Africa; 2 National Health Laboratory Service, South Africa; 3 WITS Diagnostic Innovation Hub, Johannesburg, South Africa; 4 Department of Virology, School of Medicine, Sefako Makgatho Health Sciences University, Pretoria, South Africa; 5 Division of Immunology, Department of Pathology, Faculty of Health Sciences, University of Cape Town, Cape Town, South Africa

**Keywords:** H3Africa, biobanking, nucleic acid storage, room temperature storage, dried blood spots

## Abstract

**Background:**

Maintaining nucleic acid integrity during transport and storage is important for downstream diagnostic and research applications. Innovative solutions are needed to reduce the requirement for costly cryopreservation in low- and middle-income countries.

**Objective:**

To assess the feasibility of nucleic acid storage at a range of temperatures using commercially available storage solutions and as dried blood spots (DBS).

**Methods:**

Total nucleic acids were extracted from 50 residual blood samples sourced from a routine haematology laboratory in Johannesburg, South Africa between 01 March 2021 and 15 December 2023. DNA and RNA aliquots were stored in commercial storage media and as DBS at a range of temperatures (–80 °C, 2 °C – 8 °C, room temperature [RT] and 40 °C – 50 °C) for 24 h, 96 h, 30 days and 90 days. Post-storage nucleic acid concentration and integrity were measured.

**Results:**

Four DNA and four RNA aliquots (stored complexed to beads and in commercial storage solution) and three DBS prepared from 50 whole blood samples were analysed. Nucleic acids stored at RT and at 2 °C – 8 °C maintained acceptable concentration and integrity tested using TapeStation Bioanalyser with a DNA integrity number median score of 7.5 (RT) and 7.4 (2 °C – 8 °C) for both beads and storage solutions, and DNA integrity number (acceptable range above 7). RNA integrity was moderate, with a median RNA integrity range of 3.1–3.9 (acceptable range above 5). Samples stored at 40 °C – 50 °C showed extensive degradation with no bands produced on gel electrophoresis. Dried blood spot yielded low RNA concentrations (median 1.33 ng/μL, range 0.08 – 4.63 ng/μL).

**Conclusion:**

Commercial storage solutions, beads and DBS offer a feasible alternative to cryopreservation for the storage of nucleic acids at ambient temperature.

**What this study adds:**

This study is a proof-of-concept study which investigates the feasibility of nucleic acid storage and transport at RT in Africa.

## Introduction

The Human Heredity and Health (H3) Africa consortium aimed to increase capacity for genetic research on the continent.^1,2^ H3Africa has supported a range of projects which have studied non-communicable diseases such as diabetes mellitus and cancer, and host determinants of infectious diseases such as malaria, mycobacterial infection and HIV infection and, more recently, severe acute respiratory coronavirus-2 (SARS-CoV-2) infection.^3,4^ A component of the project involved creating three state-of-the-art repositories which provided storage for nucleic material from over 51 projects from 27 countries across the continent.^5^ A major cost driver for the transport and storage of DNA and RNA for H3Africa was the requirement for low and ultra-low temperature storage of nucleic acids, particularly because some of the clinical sites were remote from the biobanks.^6^ Clinical sites in many African nations lack the downstream analytical capabilities and infrastructure needed for genomic research and molecular diagnostics.^7^ Specialised labs with advanced sequencing platforms and specialised molecular diagnostic tools are usually located far from sample collecting sites.^8^

Whole blood can be transported for short distances for immediate processing, but in areas with limited resources, extracted nucleic acids may be an alternative sample for long-distance transportation.^9,10^ Several hours after collection, whole blood, which is naturally unstable, releases nucleases that degrade DNA and RNA.^11,12^ Temperature fluctuations and continued exposure to high temperatures accelerate nucleic acid degradation in whole blood samples.^13,14,15^ Haemolysis and the presence of haemoglobin may interfere with polymerase chain reaction applications.^13,16^ Whole blood is categorised as a Category B biological substance under international shipping laws, requiring strict biosafety measures during transportation.^17^ Access to extraction facilities may be more widespread than access to sequencing and polymerase chain reaction facilities.^13^

The presence of a reactive hydroxyl group in RNA makes it susceptible to destruction, whereas DNA is relatively stable.^18^ The double-stranded helical structure of DNA provides natural resistance, with the complementary base pairing offering protection against single-strand breaks. When nucleic acids are complexed with protective agents or adsorbed onto solid supports, they can withstand temperature fluctuations that would degrade whole blood samples rapidly.^19,20^ This stability is particularly significant in African environments, where average temperatures often increase beyond 30 °C and in some places can even reach 40 °C – 50 °C.^6,21,22,23,24,25^ Nucleic acid extraction prior to transportation to regional and central facilities is an alternative to manage pre-analytical factors for downstream diagnostic and research genetic applications.^26^ Alternatives include dried blood spots (DBS), which stabilise DNA and RNA by reducing microbial growth and enzymatic activity.^10,27^ Dried blood spots are considered non-infectious for shipping and are also exempt from many of the strict biosafety and hazardous material shipping regulations that apply to whole blood samples.^28^

Quality control measures should be implemented to assess nucleic acid integrity.^29^ The tolerance for nucleic acid degradation differs based on the molecular application.^30^ Capillary electrophoresis systems generate standardised metrics including the DNA Integrity Number (DIN), which measures the total DNA profile, and the RNA Integrity Number (RIN),^31^ which analyses ribosomal RNA band patterns with values ranging from 1 (highly deteriorated) to 10 (intact, high-quality).^32^ High-quality DNA (DIN values > 7) is usually needed for high-throughput next-generation sequencing, while some polymerase chain reaction-based applications tolerate moderate degradation (DIN 5 – 7).^33^ For RNA-based applications, including gene expression profiling and RNA sequencing, RIN values above 7 are generally required for reliable results.^31^ Laboratories can improve resource allocation, determine transport conditions that affect sample quality, and make well-informed decisions on sample acceptability for different applications by implementing regular integrity testing post shipping into practice.^12^

As part of the H3Africa biorepository project, a study was undertaken to investigate the effect of temperature on the integrity of DNA and RNA. The aim of this study was to assess the feasibility of ambient temperature transport and storage of DNA and RNA at periods up to 90 days using commercially available storage solutions and DBS in a South African laboratory.

## Methods

### Ethical considerations

This project was conducted in accordance with the Declaration of Helsinki and was approved as a sub-study by the Human Research Ethics Committee (Medical) of the University of the Witwatersrand (ethics number: M1911201). The blood samples used in this study were residual de-identified samples collected at the haematology department of the Wits Diagnostic Hub, Johannesburg, South Africa, which were sent for routine full blood counts from 01 March 2021 to 15 December 2023. All sample identifiers were removed (including labels) and samples were assigned sequential numbers (S1 – S50). As all samples were fully de-identified (no identifying information, including age, sex or location was collected), the requirement for informed consent was waived. The sample data, including nucleic acid concentration and integrity at each time-point, was stored by randomly assigned sample number on a password-protected spreadsheet.

### Study design and sample selection

This study was a laboratory-based, cross-sectional assessment of nucleic acid stability and integrity following nucleic acid extraction from 50 residual whole blood samples submitted for full blood counts to the haematology laboratory at the Wits Diagnostic Hub in Johannesburg, South Africa between 01 March 2021 and 15 December 2023, and storage in commercially available storage solutions at a range of temperatures. From the 50 residual samples, a total of 1336 DNA samples were stored with 728 complexed to DNA storage beads (Machery-Nagel, Düren, Germany) and 608 stored in DNA storage solution (Gentegra LLC, Pleasanton, California, United States). A total of 1480 RNA samples were stored, with 728 stored complexed to beads (Machery-Nagel, Düren, Germany) and 752 stored in RNA storage solution (Thermo Scientific Inc., Waltham, Massachusetts, United States). A total of 546 DBSs were prepared.

Freshly collected whole blood samples in vacutainers containing ethylene diamine tetra-acetic acid (Becton Dickinson, San Jose, California, United States) were selected at random.

These samples are analysed routinely within 4 h of collection and are stored at room temperature (RT) immediately following full blood count analysis. Extraction for all the samples was performed within 4 h of sample collection and the samples were immediately de-identified and assigned study numbers to ensure patient confidentiality, after which nucleic acid extraction and DBS spotting was performed.

### Sample processing

Two 50 μL spots of whole blood from each blood sample were immediately pipetted onto two Whatman 903 filter DBS cards (Sigma-Aldrich, Saint Louis, Missouri, United States), allowed to air-dry for 3 h and then placed inside storage bags and stored at different temperatures (–80 °C, 2 – 8 °C, 18 °C – 25 °C and at 40 °C – 50 °C) and storage periods (24 h, 96 h, 30 days and 90 days). Total nucleic acids (DNA and RNA) were extracted from 200 μL of whole blood using an automated system (Hamilton Microlab Star Let M™ instrument (Hamilton, Reno, Nevada, United States) and a NucleoMag™ Pathogen kit (Machery-Nagel, Düren, Germany), and RNA from DBS using a PAXgene Blood miRNA Kit (Qiagen, Mettmann, Hilden, Germany), both according to the manufacturer’s protocol.

### Storage solutions

DNA and RNA were stored using a solution-based preservative as well as complexed to adsorption beads. Extracted DNA (10 μL) was pipetted into the GenTegra-DNA solution (Gentegra LLC, Pleasanton, California, United States), and allowed to air-dry in a biosafety cabinet for 24 h; then stored at four temperature ranges (–80 °C, 2 °C – 8 °C, 18 °C – 25 °C and 40 °C – 50 °C), according to the manufacturer’s protocol. Ambion® RNA storage solution (Thermo Scientific Inc., Waltham, Massachusetts, United States) was mixed with 10 μl of extracted RNA and immediately stored at four temperatures (–80 °C, 2 °C – 8 °C, 18 °C – 25 °C and at 40 °C – 50 °C). Adsorption beads (Machery-Nagel, Düren, Germany) were mixed with 10 μl of extracted DNA and RNA and stored immediately. When required, stored aliquots were vortexed, and placed on a magnetic separator for 5 min to remove the beads prior to downstream analysis.

Samples were stored at –80 °C inside the Thermo Scientific TSU series ultra-low freezer (Thermo Scientific Inc., Waltham, Massachusetts, United States), at 2 °C – 8 °C inside a laboratory fridge (Defy, Denver, Durban, South Africa), at controlled RT (18 °C – 25 °C) and at 40 °C – 50 °C inside a Hettich Labotec Hettcube 600R Incubator (Hettich, Kirchlengern, Germany).

### Gel electrophoresis

A 0.8% agarose gel was prepared in 1X Tris-acetate-ethylene diamine tetra-acetic acid (TAE) buffer and stained with 1.0 g/mL ethidium bromide (Bio-Rad Laboratories, Hercules, California, United States). Five μL DNA samples were mixed with 5 μL loading dye and loaded into wells of the gel. Electrophoresis was performed at 110 voltage for 1 h. DNA bands were visualised using a Bio-Rad gel-doc system (Bio-Rad Laboratories, Hercules, California, United States) equipped with ultraviolet transillumination.

### Nucleic acid quantification

DNA and RNA concentrations were measured in duplicate using a NanoDrop 1000 spectrophotometer (Thermo Scientific Inc., Waltham, Massachusetts, United States). One μL of stored DNA or RNA was pipetted onto the pedestal of the instrument and measurement was initiated to calculate the concentration and purity of the nucleic acid based on the absorbance at 260 nm and 280 nm.

### Integrity testing

Reagents were allowed to equilibrate at RT and vortexed before use. For RNA samples, a 1:5 dilution was prepared with RNA Sample Buffer (Agilent, Santa Clara, California, United States), and samples were heat denatured before loading onto the TapeStation™ (Agilent, Santa Clara, California, United States). For DNA samples, a 1:10 dilution was prepared with genomic DNA sample buffer (Agilent, Santa Clara, California, United States). The RIN and DIN were determined using the appropriate ScreenTape™ consumables (Agilent, Santa Clara, California, United States) and analysis software (Agilent, Santa Clara, California, United States).

### Data analysis

Data were recorded on a password-protected Excel spreadsheet. If appropriate, non-parametric data were normalised. Nucleic acid concentration was expressed in ng/μL and the DNA and RNA integrity numbers were expressed as DIN and RIN. A student *t*-test was utilised to calculate differences before and after storage in variables which were normally distributed and a Mann-Whitney analysis was undertaken for variables which were non-parametric. Analysis was performed utilising GraphPad Prism Version 10.3.0 (Insight Partners, New York City, New York, United States). A *p*-value < 0.05 was considered statistically significant.

## Results

### Samples included

Fifty samples were randomly selected. Total nucleic acids were extracted, and aliquots of DNA and RNA were prepared. In total, 1336 DNA samples were stored at four different temperatures for four different time periods and a total of 1480 RNA samples were stored at four different time periods at four temperatures (Table 1). A total of 546 DBSs were prepared. Regardless of the concentrations measured by the NanoDrop (Thermo Scientific, Waltham, Massachusetts, United States), samples that were preserved at 40 °C – 50 °C and did not show any identifiable bands on gel electrophoresis were not included in the total concentration analysis.

**TABLE 1:** Summary of samples collected and stored by temperature and storage solution, 01 March 2021 to 15 December 2023, Johannesburg, South Africa.

Storage solution	Temperature			
	−80 °C	2 °C – 8 °C	RT	40 °C – 50 °C
DNA Adsorption beads (Machery-Nagel, Düren, Germany)	186	180	181	181
RNA Adsorption beads (Machery-Nagel, Düren, Germany)	182	182	182	182
GenTegra-DNA solution (Gentegra LLC, Pleasanton, California, United States)	152	152	152	152
Ambion™ RNA storage solution (Thermo Scientific Inc., Waltham, Massachusetts, United States)	188	188	188	188
Whatman 903 filter DBS cards (Sigma-Aldrich, Saint Louis, Missouri, United States)	-	182	182	182

RT, room temperature.

### Nucleic acid concentration

**FIGURE 1:**
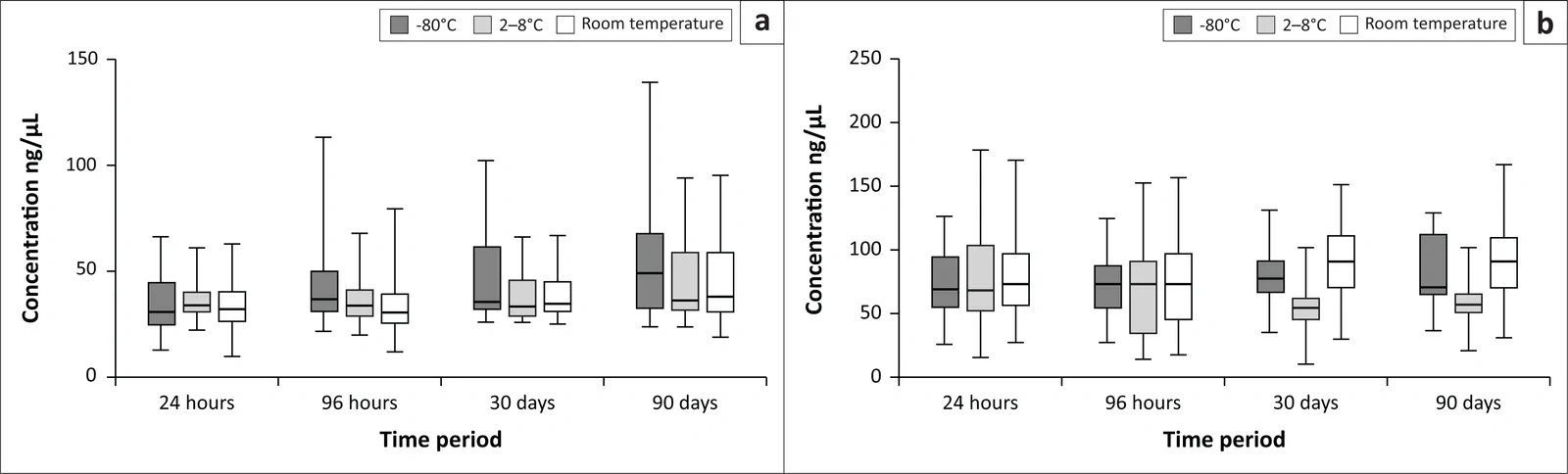
DNA concentrations from samples stored in GenTegra DNA storage solution (a) and complexed to Nucleomag™ DNA adsorption beads (b) stored at –80 °C, 2 °C – 8 °C and room temperature for 24 h, 96 h, 30 days and 90 days from 01 March 2021 to 15 December 2023, Johannesburg, South Africa.

**FIGURE 2:**
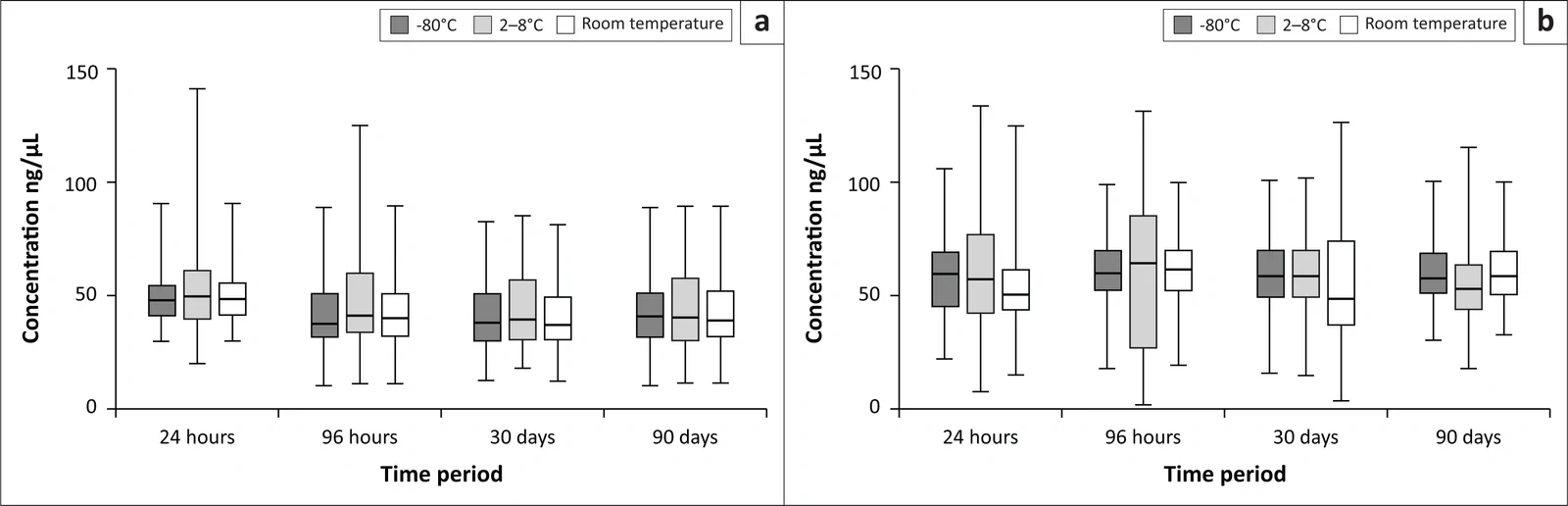
DNA concentrations from samples stored in ThermoFisher RNA storage (a) solution and complexed to RNA adsorption beads (b) stored at –80 °C, 2 °C – 8 °C and room temperature for 24 h, 96 h, 30 days and 90 days from 01 March 2021 to 15 December 2023, Johannesburg, South Africa.

There was no statistically significant difference in DNA concentration when samples were stored at –80 °C, 2 °C – 8 °C and RT in GenTegra-DNA storage solution (Gentegra LLC, Pleasanton, California, United States) (Figure 1, *p* = 0.3967) or complexed to adsorption beads (Figure 1, *p* = 0.0635). Similarly, there was no difference in RNA concentration when samples were stored at –80 °C, 2 °C – 8 °C and RT Ambion™ RNA storage solution (Thermo Scientific Inc., Waltham, Massachusetts, United States) (Figure 2, *p* = 0.5898) or complexed to RNA storage beads (Machery-Nagel, Düren, Germany) (Figure 2, *p* = 0.9131).

Only 10 samples stored at 40 °C – 50 °C had recordable DNA concentrations when stored at 40 °C – 50 °C and all samples stored at 40 °C – 50 °C failed to produce a band on gel electrophoresis (Online Supplementary Figure 1, Online Supplementary Figure 2, Online Supplementary Figure 3, and Online Supplementary Figure 4). Samples stored complexed to beads yielded statistically significant higher concentrations of nucleic acid at all temperatures than those in storage solution for both DNA (median concentration 75.35 ng/μL vs 39.33 ng/μL, *p* < 0.001) and RNA (59.66 ng/μL vs 44.91 ng/μL, *p* < 0.001).

**FIGURE 3:**
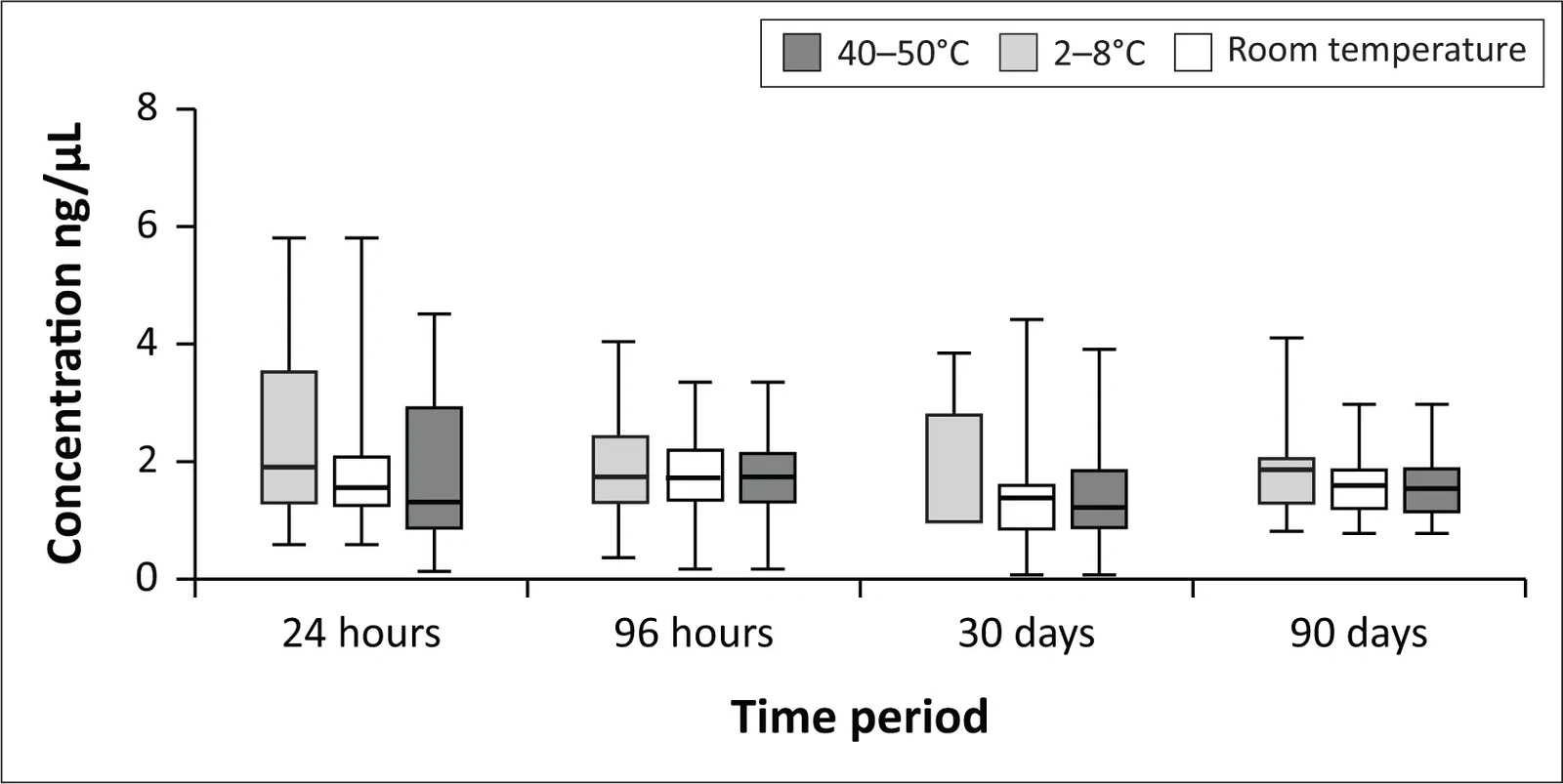
RNA concentrations from samples stored in dried blood spots at 2 °C – 8 °C, room temperature and 40 °C – 50 °C for 24 h, 96 h, 30 days and 90 days from 01 March 2021 to 15 December 2023, Johannesburg, South Africa.

The concentrations of RNA extracted from DBS were 1.5 ng/μL (range: 0.50–5.90) in samples stored at RT, 1.70 ng/μL (range: 1.03–5.60) in samples stored at 2 °C – 8 °C and 1.33 ng/μL (range: 0.08–4.63) in samples stored at 40 °C – 50 °C. Concentrations did not decline across all timepoints (Figure 3, *p* = 0.0985).

### Nucleic acid integrity

A subset of samples (*n* = 128) was selected for integrity assessment because of the high cost of the reagents required for DIN and RIN analysis. Two samples were selected at random for this study, and the nucleic acid integrity of one aliquot from each sample for each condition (temperatures and time) was analysed on the Agilent bioanalyser (Agilent, Santa Clara, California, United States). DNA integrity numbers for these samples showed consistently acceptable quality DNA (DIN 7 – 8) when stored at RT (median score 7.5, range: 7.2–7.9), 2 °C – 8 °C (median score: 7.4, range: 7.3–7.8) and −80 °C (median score: 7.6, range: 7.1–8.0) at all time points and poor DIN (2–3) when stored at 40 °C – 50 °C (median score: 2.5, range: 2.1–3.0) for all time points. RNA integrity was moderate with a score between 3 and 4 for samples stored both in beads and in storage solution at RT (median score: 3.1, range: 3.0–3.6), 2 °C – 8 °C (median score: 3.9, range: 3.1–3.8) and −80 °C (median score: 3.6, range: 3.2–3.9) at 24 h, 96 h, 30 days and 90 days.

Samples stored at 40 °C – 50 °C showed a high level of degradation at all time points (RIN median score 0, range 0 – 2). RNA Integrity Number values for RNA extracted from DBS did not produce any measurable values.

## Discussion

Storage solution and bead storage stabilise both DNA and RNA at ambient temperature for periods up to 90 days, offering a viable alternative to cryopreservation in resource-limited settings. Although the yields were low, DBS effectively preserved RNA concentration even when stored at high temperatures. The DBS RIN could not be measured accurately because the median concentration 1.33 ng/μL (range: 0.08 ng/μL – 4.63 ng/μL) of RNA obtained from DBS was below the practical sensitivity range (~5 ng/μL – 500 ng/μL) of the standard TapeStation RNA ScreenTape test, which needs detectable 18 S/28 S ribosomal RNA peaks for RIN computation.^34^ The absence of RIN may not indicate accurately the utility of RNA from DBS for downstream applications such as transcriptome analysis since RIN mostly reflects the integrity of total RNA, of which messenger RNA makes up just a tiny fraction.^35^

The aim of the H3Africa project is to investigate the genetic predictors of health in African populations.^5,6,36,37^ Biospecimens were collected from countries across the continent, some of them remote from the three biorepositories in Western, Eastern and Southern Africa.^5^

Additional delays, including border delays in customs made maintaining biospecimens at controlled ambient temperature difficult, particularly when temperatures may reach above 40 °C in some countries.^6^ Shipping on dry ice is costly and not feasible for large sample volumes.^6^

To replicate these settings, we examined sample preservation at high temperatures using four different storage solutions. Of the 50 samples stored at temperatures above 40 °C, no samples produced a band on gel electrophoresis although DNA concentrations could be measured in 10 samples. Studies of DNA integrity at high temperatures using similar storage solutions have been contradictory. A study which assessed the integrity of genomic DNA stored in GenTegra^TM^ at 56 °C for seven months found that the integrity of genomic DNA was preserved when tested in downstream sequencing applications^38^ and bacterial DNA stored at 50 °C for 64 weeks showed equivalent performance to storage at –30 °C in downstream applications with maintenance of the cycle threshold.^39^ Storage at higher temperatures (76 °C) conversely showed complete DNA degradation after one week.^40^ Degraded DNA can produce erroneous concentration measurements,^41,42^ emphasising the importance of multiple quality control checks and the adequate use of different measurement systems to address potential pre-analytical factors which may impact nucleic acid analysis.^43,44^

No recordable RNA concentration could be measured in samples stored in adsorption beads or RNA storage solution at 40 °C – 50 °C, although previous studies with GenTegra^TM^ showed acceptable RNA storage in RNA extracted from formalin-fixed paraffin-embedded lymph nodes for two weeks at 56 °C.^45^ The poor performance in this study may reflect the original sample type or the prolonged storage time. Low concentrations of RNA were obtained from DBS stored at 40 °C – 50 °C for 90 days. This may indicate that DBS are an alternative when high temperatures cannot be avoided, although the low concentration may not be useful for all applications.

Room temperature storage and storage at 2 °C – 8 °C maintained DNA and RNA concentration across the period of the study (from 24 h to 90 days) in samples stored in all commercial media, although concentrations obtained were consistently significantly higher for samples stored complexed to beads. GenTegra™ DNA storage solution performed equivalently to storage at –20 °C,^46^ and another study showed that small amounts of DNA (0.2 ng – 1 ng) could be preserved in the GenTegra^TM^ matrix at RT for at least a year without any detectable loss of integrity or impact on performance in further molecular assays.^23^ Comparable results have been seen in GenTegra^TM^ storage of bacterial DNA stored at 25 °C when compared with controls stored at –30 °C.^39^ A study which investigated the effects of GenTegra^TM^ RNA storage on gene expression profiling and RNA quality with storage at RT for more than five days measured RIN values > 7.^47^

In our study, storage of nucleic acids complexed to beads yielded consistently higher concentrations and appropriate for ambient temperature storage – a finding which is consistent with other studies.^48^ Adsorption beads provide protection against enzymatic degradation and other environmental stresses, especially in the dry state,^49^ with relatively simple recovery.^50^

Care should, however, be taken as yield may be affected by incomplete recovery or lower binding efficiencies highlighting the need for continuous quality assessment,^50^ especially for RNA which is inherently less stable.^51^ RNA is naturally less stable than DNA, requiring more comprehensive complementary stabilisers during storage.^18,51^

RNA integrity may reduce over time even in cryopreserved samples^52^ and commercial storage solutions are costly with limited access in countries in the Global South.^53^ Dried blood spots are an attractive alternative for both DNA and RNA storage, although yields may be low.^10,35,54^ Sequencing has been performed on DNA extracted from dried spots, especially in neonatal screening programmes and the utility of the DBS in RNA applications, including HIV-1 diagnosis, is being recognised increasingly.^10,28,35,54,55,56^ The development of standard operating protocols is essential to maximise the efficiency of storage and to ensure that quality control is maintained.^57^

### Limitations

This study had several limitations. It was only possible to investigate four different storage solutions and further studies will consider other commercially available solutions. Sample integrity was investigated at 90 days, and it is possible that there would be more extensive deterioration in samples stored at longer intervals. Factors which include humidity, the baseline nucleic acid concentration, the presence of host factors which may reduce or affect nucleic acid integrity, and the time between sample collections, were not measured. Baseline nucleic acid integrity metrics (DIN and RIN) were not measured in freshly extracted samples prior to storage. As a result, the study did not quantify the initial quality of the starting material directly, and all integrity assessments reflect relative changes across storage conditions and time points rather than absolute degradation from baseline. Downstream applications of the nucleic acids were not investigated extensively. This study does, however, indicate that it is feasible to transport and store nucleic acids using commercially available storage media at ambient temperature over the short term.

### Conclusion

This study confirms that long-term ambient temperature storage of both DNA and RNA is feasible over 90 days when storage solutions like adsorption beads, DNA and RNA stabilising media and DBSs are employed. RNA could be extracted from DBSs, albeit at lower concentrations, even at temperatures exceeding 40 °C, providing a low-cost and accessible alternative where temperature control is not possible.
